# Estimating the Timing of Cognitive Operations With MEG/EEG Latency Measures: A Primer, a Brief Tutorial, and an Implementation of Various Methods

**DOI:** 10.3389/fnins.2018.00765

**Published:** 2018-10-25

**Authors:** Heinrich René Liesefeld

**Affiliations:** ^1^Department Psychologie, Ludwig-Maximilians-Universität München, Munich, Germany; ^2^Graduate School of Systemic Neurosciences, Ludwig-Maximilians-Universität München, Munich, Germany

**Keywords:** magnetoencephalography (MEG), electroencephalography (EEG), component latency, mental chronometry, event-related potential/field (ERP/ERF)

## Abstract

The major advantage of MEG/EEG over other neuroimaging methods is its high temporal resolution. Examining the latency of well-studied components can provide a window into the dynamics of cognitive operations beyond traditional response-time (RT) measurements. While RTs reflect the cumulative duration of all time-consuming cognitive operations involved in a task, component latencies can partition this time into cognitively meaningful sub-steps. Surprisingly, most MEG/EEG studies neglect this advantage and restrict analyses to component amplitudes without considering latencies. The major reasons for this neglect might be that, first, the most easily accessible latency measure (peak latency) is often unreliable and that, second, more complex measures are difficult to conceive, implement, and parametrize. The present article illustrates the key advantages and disadvantages of the three main types of latency-measures (peak latency, onset latency, and percent-area latency), introduces a MATLAB function that extracts all these measures and is compatible with common analysis tools, discusses the most important parameter choices for different research questions and components of interest, and demonstrates its use by various group analyses on one planar gradiometer pair of the publicly available Wakeman and Henson (2015) data. The introduced function can extract from group data not only single-subject latencies, but also grand-average and jackknife latencies. Furthermore, it gives the choice between different approaches to automatically set baselines and anchor points for latency estimation, approaches that were partly developed by me and that capitalize on the informational richness of MEG/EEG data. Although the function comes with a wide range of customization parameters, the default parameters are set so that even beginners get reasonable results. Graphical depictions of latency estimates, baselines, and anchor points overlaid on individual averages further support learning, understanding and trouble-shooting. Once extracted, latency estimates can be submitted to any analysis also available for (averaged) RTs, including tests for mean differences, correlational approaches and cognitive modeling.

## Estimating the Timing of Cognitive Operations With Meg/Eeg Latency Measures

Remember the last time you picked apples at the grocery store? First, you had to find the shelf with the apples, then decide on the type of apple you want, then to attend to one of the apples and estimate its quality, to store this information in working memory and compare it to the alternative apples in the box; finally you had to program and execute a reaching movement etc. – this is, of course, a ridiculously coarse description of the multitude of cognitive processes involved in picking apples. Beyond any doubt, any cognitive task can be subdivided into a virtually endless number of sub-processes that unfold over time. The goal of cognitive science is to understand these sub-processes and their interplay in detail (e.g., Meyer et al., 1988). A major piece to this puzzle is the timing of sub-processes – for example, if sub-process B emerges after process A, B cannot be the cause of A.

The research tradition focusing on the timing of sub-processes is termed *mental chronometry* (Posner, 1978). Using thoughtful experimental designs, researchers were able to disentangle many of the sub-processes giving rise to performance in cognitive tasks (Meyer et al., 1988; Medina et al., 2015). Another, complementary, approach is to estimate the timing of cognitive processes via the timing of their (probable) neuronal correlates (Meyer et al., 1988; Coles, 1989; for recent examples, see Hyun et al., 2009; Töllner et al., 2012; Fortier-Gauthier et al., 2013; Ruhnau et al., 2013; Liesefeld et al., 2014; Dell’Acqua et al., 2015; Drisdelle et al., 2016; Grubert and Eimer, 2016; Liesefeld et al., 2017; Ruhnau et al., 2017; Xie and Zhang, 2018). The validity of this latter approach, of course, crucially depends on whether the examined component is indeed a valid correlate of the cognitive process of interest and the amount and quality of evidence supporting this validity varies strongly between components and interpretations of these components.

For example, the N2pc component of the event-related potential is a negativity at posterior MEG/EEG recording sites contralateral to an attended object. Whether it reflects the allocation of attention toward this object, the suppression of objects on the other side of the display or a general bias in attentional resources are heavily discussed questions (Luck and Hillyard, 1994a,b; Eimer, 1996; Hopf et al., 2000; Luck, 2012). Nevertheless, most contestants in this discussion would agree that the N2pc is somehow related to attentional dynamics and interpreting the timing of the N2pc to reflect the timing of attention shifts is therefore relatively save. N2pc timing can thus be used to measure how long it takes until certain objects draw spatial attention, which becomes particularly interesting if a task induces multiple shifts of attention (Woodman and Luck, 1999; Hickey et al., 2006; Grubert and Eimer, 2016; Liesefeld et al., 2017). Relatedly, the time at which motor-cortex activity contralateral to the responding hand rises from baseline (lateralized readiness potential, LRP) is a quite uncontroversial marker of the timing of motor preparation (e.g., Coles, 1989; Osman et al., 1992; Miller et al., 1998; Töllner et al., 2012).

Analogous points can be made for many event-related potential/event-related field (ERP/ERF) components (e.g., Verleger et al., 2005; Ruhnau et al., 2013, 2017; Liesefeld et al., 2016; Xie and Zhang, 2018). For ease of reading, the present article refers to ERP/ERFs throughout, but the latency-extraction methods are applicable to any temporally resolved correlate of cognitive processes, including other data like pupillary light response (Mathôt and Van der Stigchel, 2015) and fNIRS (Ferrari and Quaresima, 2012) and results of other preprocessing techniques of MEG/EEG data like decomposition techniques (independent component analysis, source localization, etc.), time–frequency analysis (Cohen, 2014), machine learning, and combinations thereof (Fahrenfort et al., 2017, 2018; Foster et al., 2017).

## Three Classes of Latency Measures

**FIGURE 1:**
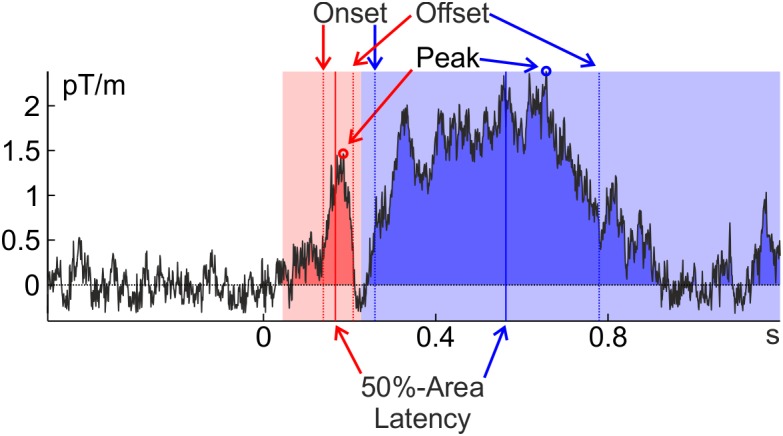
Extraction of several latency measures (peak latency, 30%-amplitude [on-/offset] latency, and 50%-area latency) from a representative individual average of the Wakeman and Henson (2015) data (famous faces at the planar gradiometer pair MEG0712 + 713 of Subject 2). An early, transient component is marked in red and a later, broad component is marked in blue. The time windows in which the peaks were searched and that confine the areas are indicated in light red and light blue; the component areas are indicated in darker red and blue. Close inspection of the graphs reveals that all latency measures incur the risk of being confounded by noise or other components (but see below for some strategies to ameliorate these potential confounds). Note that the ERF was baseline corrected.

Given the strong interest of cognitive psychologists in the timing of cognitive events and the obvious advantages of taking the timing of established neuronal markers into account, it is surprising that most research has focused on the amplitude of components instead of their latency. One reason for this issue might be that the easiest and most widely used measure of component latency – the time when a component reaches its maximum (*peak latency*) – is easily corrupted by neuronal and measurement noise (Luck, 2005; Kiesel et al., 2008). An algorithm looking for the time point with the maximal value will often pick a high-frequency noise deflection riding on top of the actual component. This noise deflection may or may not coincide with the true peak of the component; in fact, with broad components it can be far off. This is the case in Figure 1, where the peak latency of the later, blue, component is clearly an overestimation of the component latency. This is less of a problem for amplitude measures: once a reasonable temporal range of activity (e.g., ±5 ms around peak) is taken into account, high-frequency noise averages out. The quality of peak latency depends on the shape of the component – peak detection in a more transient component (with a peakier shape, like the earlier, red, component in Figure 1) is less likely to be confounded by high-frequency noise. Later components are typically broader and noise will therefore more likely influence peak latency.

A measure that is robust to high-frequency noise is *percent-area latency*. This is the time point when the component has reached a predefined percentage of its area under the curve (typically 50%). Finally, *onset latency* is the time when the component has reached some pre-defined percentage of its amplitude (e.g., 30%) and its reliability lies somewhere in between percent-area and peak latency. *Offset latency* can be defined correspondingly as the time where the component has fallen back to the pre-defined percentage, and on- and offset latency will be referred to collectively as *percent-amplitude latency*. Further latency measures that were developed for specific components are not treated here (e.g., Osman et al., 1992).

**FIGURE 2:**
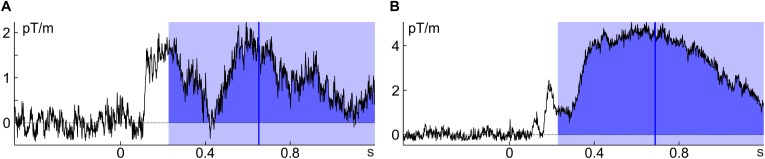
Potential issues with percent-area latency. **(A)** The time window that worked well for Figure 1 includes part of the preceding component (individual average of subject 8, unfamiliar faces); **(B)** the ERF is quite detached from baseline and thus, much of the area would typically not be considered part of the ERF (individual average of subject 3, unfamiliar faces). Note that all ERFs were baseline corrected.

For percent-area latency the definition of *area* is not necessarily straight forward: The simplest possibility is to take all activity into account that goes into the component’s direction within a predefined time window (as all activity going into the opposite direction is ignored, this is more specifically also referred to as ‘signed area’). According to this definition, area is confined by the time window, the *x*-axis (usually determined by the pre-stimulus baseline) and the ERP/ERF. To avoid missing some of the activity in some components of some individuals one would have to pick a rather broad window. This, however, incurs the risk of including activity from adjacent components or noise into the calculation and thus – depending on the data – to introduce a bias to the estimate (Figure 2A). Furthermore, components might (in contrast to the example in Figure 1) be far detached from the pre-stimulus baseline (especially if they occur rather late) and therefore much of the lower part of the area defined in this way would usually not be considered part of the component. Including this activity biases area latency toward the mean of the area window. This happens when the ERP/ERF drifts away from the pre-trial baseline into the same direction as the component (see Figure 2B).

**FIGURE 3:**
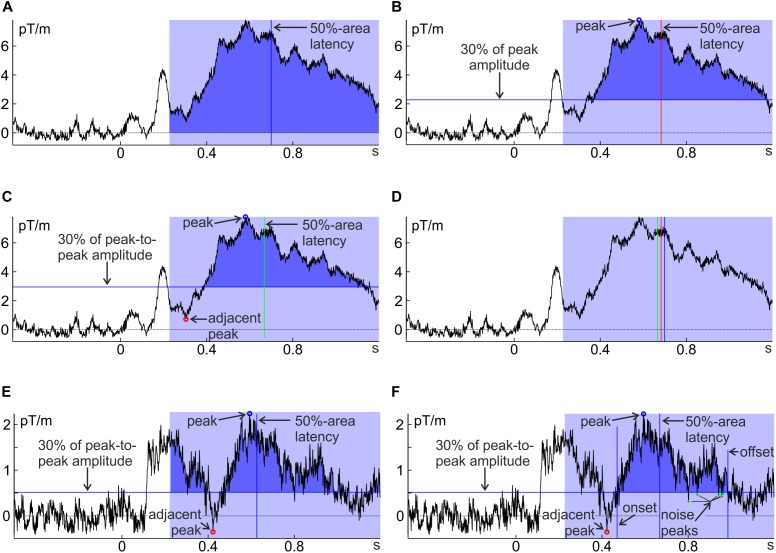
Several approaches to determine 50%-area latency, differing in the definition of component area (dark blue). **(A)** All positive values within the pre-determined interval (light blue) are added up; **(B)** only values larger than 30% of the peak amplitude are added up; **(C)** only values larger than 30% of the peak-to-peak amplitude distance above the preceding negative peak are added up, **(D)** a comparison of approaches **(A–C)** shows that latency estimates differ only little for ERFs with high signal-to-noise ratios (such as subject 15, unfamiliar faces, in **A–D**). **(E)** Same as **(C)** for another, noisier individual average (subject 8, unfamiliar faces); **(F)** same as **(E)** but with the area’s temporal boundaries set to the on- and offset of the component instead of the pre-defined analysis window. Note that toward the end of the component area there are some noise peaks crossing the 30% baseline (marked in green). These are ignored for the calculation of component offset (and therefore do not set the temporal boundaries of the area) by averaging across adjacent time points as explained in the text. Note that all ERFs were baseline corrected.

Liesefeld et al. (2016) developed several techniques to make area latency (and on-/offset latency) more robust by taking more information from the data into account: To avoid contamination by low-amplitude activity and adjacent components, one can raise/lower the baseline that constitutes the lower/upper boundary of the area (e.g., to 30% of the component amplitude; Figure 3B; see also Kiesel et al., 2008). Another useful baseline is the activity at a certain percentage in between the peak amplitude of the component of interest and an immediately adjacent component (Figure 3C). For high signal-to-noise ratios (as in the example in Figures 3A–C), these different approaches yield only slightly different estimates of component timing (Figure 3D). Adjusting the baseline will not help avoiding contamination by adjacent components of similar strength (Figure 3E); to include only the component of interest in such cases, Liesefeld et al. (2016) confined the area by the points where the component crosses the percentage-amplitude baseline for the first time before and after the peak (on- and offsets; Figure 3F). One problem with the latter approach can be that high-frequency noise crosses the baseline before the ‘real’ offset of the component. To avoid that such noise determines the end of the area window, on- and offset amplitudes can be calculated as a running average across several time points so that noise peaks are averaged with surrounding activity (an appropriately designed low-pass filter would also fulfill this function). This strongly decreases the probability that on- or offsets are determined by noise peaks (Figure 3F).

According to anecdotal evidence^1^, these techniques are robust against noise and avoid confounds with adjacent components while still taking into account the whole component, even if it is subject to substantial interindividual variability. In particular, the Liesefeld et al. (2016) method was developed to hedge against low-frequency noise (by adapting the baseline), high-frequency noise (by employing an area-latency approach and averaging amplitudes across several sampling points) and confounds by other components (by confining the area by on- and offsets). It thus holds promise to yield stable estimates of component latency even under difficult (i.e., noisy) conditions. The disadvantage of this measure lies in the number of parameters the researcher has to set; this will become more evident below where the parameters are explained in detail (some strategies will be outlined to arrive at reasonable decisions). Also note that the Liesefeld et al. (2016) adaptations of area latency (and on-/offset latency) were developed for the analysis of ERPs and that their suitability for other types of data (e.g., fNIRS) and other analysis techniques (e.g., time–frequency analysis) should be validated separately.

## Complementary Approaches to Handle Noise

Problems related to high- and low-frequency noise can often be ameliorated by respective filtering before extraction of the latency measure. However, filters (especially on segmented data) might introduce artifacts and often not only decrease the noise level, but also the signal (i.e., the component of interest; Luck, 2005). Furthermore, designing the right filter also comes with many decisions on setting parameters (for an overview, see Widmann et al., 2014) and requires considerable experience with filter design. Thus, filter-free approaches might be preferred by some (for insightful discussions on the pros and cons of filtering and how potential pitfalls might be avoided (also in the particular case of latency estimates), see Maess et al., 2016a,b vs. Tanner et al., 2015, and VanRullen, 2011, or Tanner et al., 2016, with responses from Rousselet, 2012, and Widmann and Schröger, 2012). In general, it is of high importance to carefully extract the component of interest (i.e., improve the signal-to-noise ratio) without distorting it by applying adequate pre-processing steps – such as baseline corrections, averaging, calculating difference waves (between conditions or electrodes), time–frequency decomposition, decomposition into spatio-(spectro-)temporal clusters and many more – before attempting to estimate its timing. What is adequate depends on various characteristics of the component of interest, quality of the data, and state of the art regarding the targeted component and cognitive function and is therefore not detailed here (see Luck, 2005, 2014, and Cohen, 2014 for excellent general introductions; see many other articles in this Special Issue for step-by-step guides on various techniques).

*Jackknifing* is another approach to handle noise (Miller et al., 1998; Ulrich and Miller, 2001; Stahl and Gibbons, 2004; Brisson and Jolicoeur, 2008; Kiesel et al., 2008; Smulders, 2010): Even though individual averages are likely contaminated by high- and low-frequency noise, the grand average across all subjects is less so. Thus, the best estimate of a component’s latency would be based on the grand average ERP/ERF. However, getting rid of all interindividual variability also means that no statistical tests can be employed to test for latency differences, e.g., between conditions. Jackknifing provides the best of both worlds: averages are created across all but one of the *n* individual data sets with each data set being left out once. Latencies are then extracted from each of the *n* leave-one-out grand averages. Given that much more data contributes to each of these averages (*n* – 1 times the data of individual averages, typically), any latency estimate is much less influenced by noise. Still the variance between the leave-one-out averages provides some indication of the error variance (interindividual variance in latencies) and (appropriately adjusted) statistical tests can be performed (Miller et al., 1998; Ulrich and Miller, 2001).

An obvious disadvantage of jackknifing is that there are no individual estimates of component timing and that appropriate tests must be developed for each statistical test (e.g., Miller et al., 1998; Ulrich and Miller, 2001; Stahl and Gibbons, 2004), rendering the technique rather inflexible. This problem can be resolved using a technique to restore such individual estimates from jackknife estimates (Brisson and Jolicoeur, 2008; Smulders, 2010).

## *latency.m*: A Matlab Function for Latency Extraction

The following, will demonstrate how all these latency estimates (and versions thereof) are extracted from group data, using the MATLAB (The Mathworks, Natick, MA, United States) function *latency.m* (the most current version is available here^2^), so that they can be submitted to statistical tests.^3^ This function can be used with output from common MATLAB-based analysis toolboxes (EEGlab, Delorme and Makeig, 2004; Fieldtrip, Oostenveld et al., 2011) or data converted to MATLAB (e.g., from BrainVision Analyzer, BrainProducts, Munich, Germany). It requires the Signal Processing Toolbox (The Mathworks, Natick, MA, United States). All of the following examples will be done on publicly available MEG data collected by Wakeman and Henson (2015) as preprocessed by Robert Oostenveld^4^. In particular, the examples make use the event-related averages in *timelock_x_cmb.mat* (where *x* stands for *faces*, *famous*, *scrambled*, and *unfamiliar*). All code needed to generate raw versions of the figures shown above and the example analyses described below from the Wakeman-and-Henson data is deposited at figshare^5^. Note that the purpose of this article is *not* to introduce or validate any new method, but to provide a tutorial example of how to extract latency estimates for running group analyses from already preprocessed data, including statistical tests of latency differences between conditions.

### Basic Input and Output

The following describes the various input parameters (see Table 1 for a summary) and resulting outputs of *latency.m*. A list of all parameters with a short description, including the default values, as well as all possible outputs is contained in the MATLAB function and displayed using *help latency*. The function expects two input arguments: the individual, preprocessed averages (*avgs*) and a configuration structure^6^ (*cfg*; similar to Fieldtrip, Oostenveld et al., 2011). So, a valid call to the function is *res = latency(cfg, avgs)*. The input *avgs* is a Subjects × Channels × Time matrix with the individual averages or a structure that contains such a matrix as the fields ‘*data*’ (EEGlab) or ‘*individual*’ (Fieldtrip); one can also specify the name of the data field via *cfg.datafield*. The function also recognizes a cell array of structures (one structure for each subject) with the data stored in the field ‘*avg*’ (such as those produced by the Oostenveld script). The only parameter that must be specified is the sign of the component by setting *cfg.sign* to either ‘*1*’ or ‘-*1*’ (or to ‘*pos*’/‘*neg*,’ alternatively). One should additionally indicate which type of latency estimate to extract, for example, *cfg.extract* = ‘*peakLat’*. With these settings, *latency.m* returns the latency of the local maximum in the indicated direction (peak latency) with data averaged across all channels. Usually, one would like to restrict the temporal search space by setting *cfg.peakWin* according to the temporal extent of the component (with some leeway to account for individual differences). Channels of interest are selected via *cfg.chans*. Indicate either multiple channels if an unweighted average across these channels is desired (which is less likely for ERF than for ERP analyses) or only one channel (that may contain a weighted combination of original channels, e.g., the result of a decomposition). To include only specific subjects, select them via *cfg.subs*. Time points, channels, and subjects are by default addressed by their positions in the matrix (indices); *cfg.peakWin* expects start and end points, *cfg.chans* and *cfg.subs* expect all indices (to allow for choosing non-adjacent channels and subjects, which would not usually make sense for *cfg.peakWin*). Alternatively, subjects can be addressed by their designation in the experiment if, additionally, a list of subject numbers is provided as *cfg.subNum* (array of integers). The same is possible for channels by specifying *cfg.chanNames* (cell array of strings) and times by specifying *cfg.times* (array of numbers). Notably, if *cfg.times* is specified, all parameters specifying times are interpreted as and latency estimate(s) are returned in the units of *cfg.times* (ms or s) instead of sampling points. *cfg.subNum, cfg.chanNames* and *cfg.times* must be the same order as in the Subjects × Channels × Time data matrix.

**Table 1:** Fields of the configuration structure (*cfg*). See the text for details.

Name	Description	Options	Default
extract	(List of) measures to extract	‘all’ or any combination of the following: ‘mean,’ ‘peakLat,’ ‘onset,’ ‘offset,’ ‘width,’ ‘areaLat,’ ‘peakAmp,’ ‘peak2peak,’ ‘percAmp,’ ‘area’	‘all’
aggregate	How to combine the data	‘individual,’ ‘GA,’ ‘jackMiller,’ ‘jackSmulders’	‘individual’
fig	To plot ERPs and latency estimates	True, false, 1, 0	False
subs	Subjects to include into the analysis	Indices/subject numbers or logical filter	All
subNum	List of subject numbers	Subject numbers in correct order: vector of length(avgs,1)	
chans	Channels to average across	Indices or channel names	All
chanName	List of channel names	Channel names (strings) in correct order: vector of length(avgs,2)	
peakWin	Search window for detecting the peak	Start and end of search interval: vector with two elements	Whole range
meanTime	Window for extracting mean amplitude	Start and end of averaging interval: vector with two elements	peakWin
times	Information on the time scale of the data	Individual time points: vector of length(avgs,3); alternatively, start, end and sampling rate: vector with three elements	
peakWidth	Determines averaging window for peak amplitude	Time around peak (±peakWidth)	Five sampling points/∼5 ms
cWinStart	Point from where the counter-peak is searched	‘peak’ or ‘peakWin’	‘peakWin’
cWinWidth	Width of the counter-peak search interval	Time before (negative values) or after (positive values) cWinStart: single number	
cWin	Search interval for counterpeak (alternative to cWinStart/Width)	Start and end of search interval: vector with two elements	
percArea	Percentage of the total area for percent-area latency	Value in between 0 and 1	0.5
percAmp	Percentage of the amplitude (peak-to-peak, if a counter peak is used)	Value in between 0 and 1	0.5
areaWin	Determines temporal boundaries for area calculation	‘peakWin,’ ‘ampLat,’ ‘fullRange’; alternatively, start and end of area: vector with two elements	‘peakWin’
areaBase	Determines one area boundary in amplitude space	‘zero’ (*x*-axis; typically pre-stimulus baseline) or ‘percAmp’	‘zero’
ampLatWin	Determines where on- and offsets are searched	‘fullRange,’ ‘peakWin,’ alternatively, start and end of search interval: vector with two elements	‘fullRange’
cBound	Determines whether counter peak is one border of the search interval for on- and offsets	True, false, 1, 0	True
warnings	Determines whether warnings are shown	True, false, 1, 0	True


If *cfg.extract* is not set, *latency.m* will extract everything it can, using default parameters where applicable. It is, however, recommended to choose one (or a few) latency measure(s) *a priori* by setting *cfg.extract*. Use tilted brackets to submit a list of desired output measures, separated by commas. Possible latency measures are peak latency (‘*peakLat*’), percent-amplitude latency before (‘*onset*’) or after (‘*offset*’) the peak, and percent-area latency (‘*areaLat*’). In addition it is possible to extract other measures that are created along the way or might be useful for other analyses or quality checks, namely mean amplitude (‘*mean*’), peak amplitude (‘*peakAmp*’), total area under the curve (‘*area*’), width of the component (offset – onset; ‘*width*’), difference in peak amplitude between the component of interest and an adjacent component of opposite polarity (‘*peak2peak*’), and the new baseline for the area boundary (‘*baseline*’). ‘*counterAmp*’ and ‘*counterLat*’ are the amplitude and the latency of the preceding (*cfg.cWinWidth* < 0) or following (*cfg.cWinWidth* > 0) adjacent peak (referred to as *counter peak* here). Furthermore, there are several Booleans that indicate for each subject whether a local peak was found (‘*foundLocal*’)^7^, whether on- and offsets were found *(‘foundOn,’ ‘foundOff*’) and whether a point dividing the area into the desired percentage (area latency) was found (‘*foundArea*’). When a single output measure is requested, the output *res* is a vector with one value for each participant, otherwise *res* is a structure with the respective fields for each output measure. An additional output can be requested (*cfgNew*), which contains all information on the final settings, including parameters that were not set at call and were filled with default values, and the field *ann*, which contains some in depth information on the extracted measures that might be useful for understanding and reporting the results.

Peak latency (*peakLat)* is extracted from the desired spatiotemporal analysis window without any additional parameters. Percent-amplitude latency (*onset* and *offset*) and percent-area latency (*areaLat*) require some parametrizing, which will be detailed right away.

### Percent-Amplitude Latency (On- and Offset)

Percent-amplitude latency is the time point at which the component has reached a certain percentage of its peak amplitude (typically 50%). This percentage is set via *cfg.percAmp*. The point before the peak, where activity has reached this threshold is called *onset* and the point after the peak can analogously be referred to as *offset*. Peak amplitude (*peakAmp*; which is calculated as an intermediate step) should be an average across several sampling points to avoid contamination by high-frequency noise. Thus, a second parameter that determines the width of this averaging window is necessary. *cfg.peakWidth* is the number of sampling points to the left and right of the peak latency that is averaged and defaults to 5 sampling points or ∼5 ms (i.e., 11 sampling points or ∼11 ms are averaged). The same number of sampling points is also averaged for determining the amplitude at on- and offset (which is used for the decision whether the desired percentage of the amplitude has been reached), so that percent-amplitude latency is not contaminated by high-frequency noise (see Figure 3F). If no averaging across sampling points is desired, (e.g., because an appropriately designed low-pass filtered has already removed high-frequency noise), set *cfg.peakWidth* = 0.

As discussed above (and displayed in Figure 3) later components often do not cross the pre-stimulus baseline due to slow-wave activity or low-frequency noise, thus sometimes introducing a bias toward earlier time points in onset latency (e.g., Figure 3A). Liesefeld et al. (2016) devised a way to handle these drifts. Instead of defining percent-amplitude latency with respect to the pre-stimulus baseline, they defined it with respect to a certain percentage of the peak-to-peak amplitude difference between the component of interest and an adjacent component. This can be done in *latency.m* by setting *cfg.cWinWidth*. This parameter indicates how much before (negative values) or after (positive values) the search-window border (*cfg.cWinStart = ‘peakWin*’; default) or the peak of the component of interest (*cfg.cWinStart = ‘peak*’) the algorithm should look for the peak of the adjacent component. If *cfg.cWinWidth* is set, *cfg.percAmp* no longer refers to the percentage of component amplitude relative to the pre-stimulus baseline, but to the percentage of the peak-to-peak amplitude (e.g., *cfg.percAmp = 0.5*, will result in the time where the amplitude is in between that of the two peaks; values > 0.5 will result in times closer to the peak of interest).

*cfg.ampLatBound* is used to control the temporal extent in which the algorithm searches for percent-amplitude latencies. *cfg.ampLatBound = ‘peakWin*’ restricts this search to the search interval used to determine the component’s peak; ‘*fullRange*’ (default) does not restrict the search range. If *cfg.cWinWidth* is set and *cfg.cBound* is *true* (default), the peak of the other component determines one temporal boundary. Alternatively, *cfg.ampLatBound* can be set by hand by providing start and end times.

### Percent-Area Latency

Percent-area latency is the time point where a component has reached a certain percentage of its area (set via *cfg.percArea*; typically 50%). As discussed above, the crux is the definition of area. In the simplest (default) case, the area is confined in amplitude space by the ERP/ERF and the pre-stimulus baseline (*cfg.areaBase* = ‘*zero*’). If *cfg.areaBase* is set to ‘*percAmp*’ the desired percentage of the peak amplitude (*cfg.percAmp*) serves as a boundary in amplitude space; in a way, the baseline is moved toward the peak of the component (thus decreasing the area; see also Kiesel et al., 2008). In time, the area is confined by the indicated time window (*cfg.areaWin*, which defaults to *cfg.peakWin*, but can also be set by hand) or by the on- and offsets *(cfg.areaWin* = ‘*ampLat*’).

### Extracting Jackknife and Grand-Average Latencies

The default output of *latency.m* is one latency estimate per subject. The function can also return jackknife estimates for any output measure by setting *cfg.aggregation = ‘jackMiller.*’ For sample size *n*, the output will contain *n* jackknife estimates plus the respective grand average estimate as the last entry. These must then be analyzed with appropriate statistical tests (Miller et al., 1998; Ulrich and Miller, 2001; Stahl and Gibbons, 2004). For paired *t*-tests, the figshare folder (see footnote 5) contains a small function called *jackT.m* implementing the Miller et al. (1998) formula. Alternatively, the method of Smulders (2010) can be used by setting *cfg.aggregation = ‘jackSmulders.*’ If latencies of the grand average are needed, set *cfg.aggregation = ‘GA.*’

## Deciding on Parameter Settings

There is quite some flexibility in choosing parameters, incurring the risk of arriving at sub-optimal solutions or bogus effects (Simmons et al., 2011; Luck and Gaspelin, 2017). However, (a) under high signal-to-noise conditions most (reasonable) settings should typically converge to the same conclusions (see Figure 3D), (b) the validity of individual latency estimates can be easily verified using an in-built graphical representation (set *cfg.fig = true*), and (c) the descriptions of the various latency measures above and the further advice and the examples below point to quite a few principles that can be used to considerably restrict the parameter space *a priori*. Furthermore, using on- and offsets to constrain the area (as suggested by Liesefeld et al., 2016) will make percent-area-latency estimates quite robust against the choice of the analysis window (see Luck and Gaspelin, 2017, for advantages of analysis-window independency); that is, the increase in parameters fed into the function is likely (more than) balanced by a reduction in the arbitrariness of the choice of analysis window. In general, to avoid analyst-induced biases, suitable parameters should be identified based on data averaged across conditions where the analyst is blind to any condition differences (Luck and Gaspelin, 2017).

### Choice of Latency Estimates and Parameter Settings

Choosing the appropriate latency measure and deciding on parameter settings is a matter of expertise and depends on the data set and research question at hand. Nevertheless, a few general recommendations apply to ERP/ERFs: Due to its low reliability, peak latency should usually be avoided. It can give reasonable results for very transient components under high signal-to-noise-ratio conditions, though. Even then applying a relatively strict low-pass filter (considerably attenuating all frequencies above 30 Hz) before the peak detection is typically necessary (see also Luck, 2005). Percent-amplitude latency should be used when the hypotheses relate to the onset (or offset) of the component. A fairly low percentage of the component amplitude, e.g., 10%, would reflect the true on- or offset of the component. On the downside, a low percentage makes percent-amplitude latency prone to noise so that higher percentages are often advisable (e.g., 30%). Also note that onset latency is always biased toward the earliest component onsets (across trials and, for jackknife or grand average latencies, additionally across subjects), because these determine when the individual averages deviate from the baseline. Likewise the offset is biased by the latest component offsets. Furthermore, with slowly rising components and moderate levels of high-frequency noise, onset latency will be relatively unreliable. In most cases, 50%-area amplitude is the best choice, because it provides a reliable estimate of the median component latency (Luck, 2005). Lower or higher area percentages can be used to capture more the on- or offset of the component.

If the component of interest occurs relatively late and there is contamination with low-frequency noise or slow-wave activity, the baseline adaptation of Liesefeld et al. (2016) should be used. In most cases, using the adjacent peak as an anchor point and bounding the area by on- and offsets as suggested by Liesefeld et al. (2016), should improve the results of percent-amplitude latency and percent-area latency, because it increases the robustness against slow-wave activity and low-frequency noise and it reduces confounds with the area of other (close) components (see Figure 3). Furthermore, as it allows using more generous windows, interindividual differences in component timing are less likely to push (part of) the component out of the window for some subjects. Where to search for the adjacent peak (i.e., the specific values for *cfg.cWinStart* and *cfg.cWinWidth*) heavily depends on the observed data pattern and can likely not be fully determined *a priori*, but must be based on an inspection of the grand average across conditions (see previous section). Baseline adaptation and adjacent-peak anchor are typically unnecessary if the component of interest is well isolated during preprocessing (e.g., when the contaminating slow-wave activity is subtracted out to a large extent by calculating differences between conditions or hemispheres).

### Troubleshooting

A good way to get used to the function is to set *cfg.sign* according to the direction of the component, run *[res, cfgNew] = latency(cfg, avgs)*, read the warning messages and inspect the contents of *cfgNew*, which will tell you about the default parameters. This should already provide quite some clues on which parameters one would like to change. Warnings can be turned off by setting *cfg.warnings = false*.

One common problem is that no local peak is found and the algorithm returns one of the boundaries of the search interval (*cfg.peakWin*) instead. If this happens too often (for more than half the participants), the algorithm returns a warning. This typically indicates that the search interval is set too narrowly and, thus, increasing *cfg.peakWin* often helps. The output *res.foundLocal* indicates for each subject whether a local peak was found or not and, thus, helps to identify anomalous individual averages (e.g., not showing the component of interest or containing a high level of low-frequency noise).

Percent-amplitude latency is corrupted when the ERP/ERF does not cross the baseline before or after the peak. This is the case when the indicated percentage of the component amplitude is already reached before the onset of the search interval or activity does not fall sufficiently again after the component’s peak. The algorithm than sets the on- or offset estimate to the respective search boundary (determined by *cfg.ampLatWin*). If this happens too often (for more than half of the participants), the algorithm returns a warning. One reason might be that a slow-wave component overlays the component of interest. In this case, using the adjacent peak as an anchor point and changing the baseline as described above holds promise to considerably improve the results. If this occurs for only a few participants, their individual averages might be too noisy and should be rejected. Inspect ‘*res.foundOn*’ and ‘*res.foundOff*’ to identify such corrupted data sets.

A more general troubleshooting strategy is to set *cfg.fig = true*. This will produce a figure for each subject with a graphical depiction of the various extracted measures, baselines, and anchor points. Visual inspection of these figures will often help to identify issues with the parameters or individual averages. These figures will also help understanding what exactly the function is doing, so that inspecting them is advisable whenever there is any uncertainty regarding the latency-extraction procedure.

## Example Analysis of Group Data

### Example Data and Parameter Settings

**FIGURE 4:**
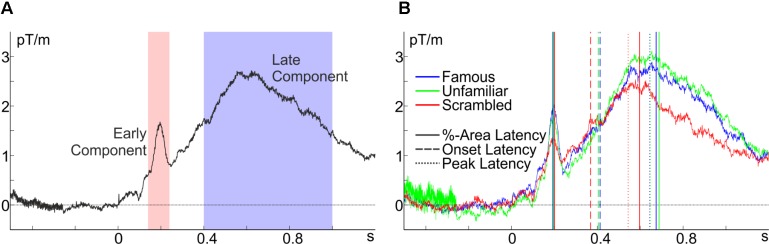
Baseline-corrected grand averages at the planar gradiometer pair MEG0712 + 713 of the Wakeman and Henson (2015) data. **(A)** Average across all conditions with peak-search windows for the early (red) and the late component (blue), and **(B)** the conditional averages with the extracted latency estimates.

As a practical example, the usage of *latency.m* will be demonstrated on the planar gradiometer pair MEG0712 + 713 of the freely available MEG data set of Wakeman and Henson (2015) as preprocessed by Robert Oostenveld (see footnote 4), which was already used in the examples above (all code and the preprocessed data are at figshare; see footnote 5). These data were collected from observers looking at scrambled or intact faces, whereby faces were either famous persons or unfamiliar to the observer (see Wakeman and Henson, 2015, for details). Inspection of the grand average across all individuals and conditions (Figure 4A) indicates that there are two prominent components: an early transient positivity and a later broad positivity. Inspection of Figure 4B indicates that the peak and median latency, but not the onset, of the latter component differs for intact and scrambled faces and that this effect is absent in the earlier component. For illustrative purposes, let us assume that these are well-characterized components, that pre-processing was adequate for extracting them, and that this is the predicted pattern of results, so that we can test them for significance using *latency.m*. For reasons outlined above, this example uses 50%-area latency with area bound by the on- and offset of the component (*cfg.areaWin = ‘ampLat*’), which are defined as 30% of the peak amplitude (*cfg.percAmp* = 0.3) relative to the adjacent negative peak. With regard to the shape of the overall ERF displayed in Figure 4A and in order to definitely include the peak of the component, but avoid the peak of the respective other component, the algorithm is set to search in the time windows 140–240 ms (*cfg.peakWin* = [0.14, 0.24]) and 400–1,000 ms (*cfg.peakWin* = [0.4, 1]) for the peak of the early and late component, respectively. The adjacent peak is searched starting 200 ms before the search window for the late component (*cfg.cWinWidth* = -0.2) and until 100 ms after the search window for the early component (*cfg.cWinWidth* = 0.1).

### Classical *t*-Tests

Indeed, confirming the initial observation, the 50%-area latency of the late component differed between intact and scrambled faces (mean difference, $\overset{¯}{d}$ = 90.2 ms), *t*(15) = 3.39, *p* = 0.004, but not between famous and unfamiliar faces, *t*(15) = 1.24, *p* = 0.234, $\overset{¯}{d}$ = 13.1 ms. Notably, this was not due to a difference in onset latency, *t*(15) = 1.20, *p* = 0.249, $\overset{¯}{d}$ = 36.7 ms, and *t*(15) = 0.50, *p* = 0.627, $\overset{¯}{d}$ = 6.7 ms, respectively. An analysis of peak latencies confirmed the area-latency results, *t*(15) = 3.51, *p* = 0.003, $\overset{¯}{d}$ = 125.8 ms, and *t*(15) = 0.15, *p* = 0.885, $\overset{¯}{d}$ = 3.8 ms, respectively. An analysis of the earlier component’s area latency with the same parameters (except for the time windows) showed no significant differences in latency between intact and scrambled faces, *t*(15) = 1.58, *p* = 0.135, $\overset{¯}{d}$ = 11.3 ms, or between famous and unfamiliar faces, *t*(15) = 0.35, *p* = 0.734, $\overset{¯}{d}$ = 2.0 ms.

### Jackknife *t*-Tests

Surprisingly, jackknife analyses of the late component’s peak latency did not confirm the pattern, *t*(15) = 1.06, *p* = 0.306, $\overset{¯}{d}$ = 77.3 ms, and *t*(15) = 0.01, *p* = 0.991, $\overset{¯}{d}$ = 0.9 ms, respectively, for the Miller et al. (1998) method, and *t*(15) = 0.94, *p* = 0.364, $\overset{¯}{d}$ = 68.2 ms, and *t*(15) = 0.06, *p* = 0.956, $\overset{¯}{d}$ = 4.4 ms, respectively, for the Smulders (2010) method. Without overstraining this serendipitous finding here, this might indicate that under certain conditions (probably with high signal-to-noise ratios and components without a clear singular peak, see Figure 4) jackknife estimates of peak latency can be inferior for detecting existing differences compared to individual peak-latency estimates. Using jackknife estimates of 50%-area latency, recovered the pattern, *t*(15) = 3.43, *p* = 0.004, $\overset{¯}{d}$ = 79.1 ms, and *t*(15) = 0.55, *p* = 0.591, $\overset{¯}{d}$ = 16.4 ms, for intact vs. scrambled and famous vs. unfamiliar faces, respectively, for the Miller et al. (1998) method, and *t*(15) = 3.36, *p* = 0.004, $\overset{¯}{d}$ = 77.6 ms, and *t*(15) = 0.48, *p* = 0.639, $\overset{¯}{d}$ = 14.3 ms, respectively, for the Smulders (2010) method.

### Reliability and Interrelation of Latency Measures

These analyses indicate that latency does not differ depending on whether the face is famous or unfamiliar. This opens the interesting possibility to use the correlation between the late component’s latencies for famous and unfamiliar faces as an index of the reliability of the various measures. This estimate of reliability was highest for area latency, *r* = 0.94, *p* < 0.001, second for onset latency, *r* = 0.86, *p* < 0.001, and worst (although still acceptable) for peak latency, *r* = 0.70, *p* = 0.002. Furthermore, the inter-correlations of the various measures of the late component’s latency might serve to gauge in how far these measures capture the same aspects of the underlying process. Area latency correlated highly with peak latency, *r* = 0.84 (corrected for attenuation, *r*_corr_ = 1), *p* < 0.001, and weaker with onset latency, *r* = 0.50 (*r*_corr_ = 0.56), *p* = 0.048, but the correlation between onset and peak latency did not reach significance, *r* = 0.21 (*r*_corr_ = 0.27), *p* = 0.436. As makes intuitive sense, area latency and peak latency pick up the same variance, which is different though somewhat related to onset latency.

## Concluding Remarks

The present article illustrated the importance of component-latency measures for cognitive theories, introduced the three most common latency measures and variants thereof and discussed their strength and weaknesses. Furthermore, it described a function that can extract all these measures from group data (*latency.m*) and applied this function to an MEG data set (Wakeman and Henson, 2015). An accompanying figshare folder (see footnote 5) contains the version of *latency.m* used here, a function for performing paired *t*-tests on jackknife data, and a MATLAB script and all dependencies for reproducing raw versions of all figures as well as all results of the example analyses. The most current version of *latency.m* can be downloaded from https://github.com/Liesefeld/latency.

## Author Contributions

HRL is the sole author and has done all the work involved in preparing and writing this manuscript.

## Conflict of Interest Statement

The author declares that the research was conducted in the absence of any commercial or financial relationships that could be construed as a potential conflict of interest.

## Footnotes

1 A formal validation using surrogate data where the true timing of components is known is beyond the scope of this tutorial introduction and must await further dedicated research. Yet, versions of this technique have arguably provided compelling results in Liesefeld et al. (2016, 2017) as well as in the examples provided here.

2 https://github.com/Liesefeld/latency

3 See the ERP Measurement Tool (available from https://github.com/lucklab/erplab/wiki/ERP-Measurement-Tool) for an alternative that provides a user interface. The main differences to the present function are that it is integrated into a particular toolbox (ERPLAB) and that the modifications of on-/offset latency and area latency suggested by Liesefeld et al. (2016) as well as jackknife estimates are not implemented.

4 https://github.com/robertoostenveld/Wakeman-and-Henson-2015

5 https://figshare.com/projects/MEG_EEG_latency/39068

6 A structure is a MATLAB data type that groups data containers called ‘fields’ (and potentially subfields), each of which can hold any type of data (see MATLAB/Language Fundamentals/Data Types/Structures in the online MATLAB documentation). This approach is preferable to differentiating parameters by their input order, because it is less prone to confusion or omission errors (switching the position of two parameters or accidentally omitting one parameter so that all following parameters are interpreted incorrectly by the function). One (among others) advantage the configuration structure has in comparison to the key + value input method [e.g., latency(avgs,‘sign,’1)] is its re-usability in several calls to the function, e.g., for applying the exact same parameters to several datasets (conditions) or changing single parameter values per call such as the analyzed (cluster of) channel(s) in *cfg.chans* (see Table 1).

7 Usually a local peak (the sample with the highest [or lowest] value that has samples with smaller [or higher] values on both sides) is returned, a global peak (one border of the analysis window that contains the highest [or lowest] value) is only returned in the rare cases that no local peak is found. See the section “Troubleshooting” below.
